# Composite Bone Cements with Enhanced Drug Elution

**DOI:** 10.3390/polym15183757

**Published:** 2023-09-14

**Authors:** Kirill Cherednichenko, Adeliya Sayfutdinova, Denis Rimashevskiy, Birzhan Malik, Andrey Panchenko, Maria Kopitsyna, Stanislav Ragnaev, Vladimir Vinokurov, Denis Voronin, Dmitry Kopitsyn

**Affiliations:** 1 Department of Physical and Colloid Chemistry, Faculty of Chemical and Environmental Engineering, National University of Oil and Gas “Gubkin University”, Moscow 119991, Russia; cherednichenko.k@gubkin.ru (K.C.);; 2 Department of Traumatology and Orthopedics, Peoples’ Friendship University of Russia, Moscow 117198, Russia; 3 Astana Medical University, Beybitshilik Street 49a, Astana 010000, Kazakhstan; 4 Russian Institute for Scientific and Technical Information “VINITI RAS”, Moscow 125190, Russia; 5 Multidisciplinary Hospital Named after Professor Kh.Zh. Makazhanov, Karaganda 100000, Kazakhstan

**Keywords:** bone cement, ALBC, PMMA, vancomycin, antibiotic release, nanocellulose, NFC

## Abstract

Antibiotic-loaded bone cement (ALBC) has become an indispensable material in orthopedic surgery in recent decades, owing to the possibility of drugs delivery to the surgical site. It is applied for both infection prophylaxis (e.g., in primary joint arthroplasty) and infection treatment (e.g., in periprosthetic infection). However, the introduction of antibiotic to the polymer matrix diminishes the mechanical strength of the latter. Moreover, the majority of the loaded antibiotic remains embedded in polymer and does not participate in drug elution. Incorporation of the various additives to ALBC can help to overcome these issues. In this paper, four different natural micro/nanoscale materials (halloysite, nanocrystalline cellulose, micro- and nanofibrillated cellulose) were tested as additives to commercial Simplex P bone cement preloaded with vancomycin. The influence of all four materials on the polymerization process was comprehensively studied, including the investigation of the maximum temperature of polymerization, setting time, and monomer leaching. The introduction of the natural additives led to a considerable enhancement of drug elution and microhardness in the composite bone cements compared to ALBC. The best combination of the polymerization rate, monomer leaching, antibiotic release, and microhardness was observed for the sample containing nanofibrillated cellulose (NFC).

## 1. Introduction

Polymethylmethacrylate (PMMA)-based antibiotic-loaded bone cement (ALBC) is widely employed in orthopedic surgery all over the world [1,2,3,4,5,6,7,8,9]. As an antibiotic carrier, ALBC provides local drug delivery at the surgical site in concentrations sufficient for the successful suppression of the infection (e.g., periprosthetic joint infection (PJI)) [2,6,9,10,11,12,13]. Moreover, ALBC application can be advantageous compared to systemic antibiotic administration since it provides higher local concentrations, lowers rates of antibiotic-associated adverse events, and decreases antimicrobial resistance via the preservation of the gastrointestinal microbiome [8]. During the last four decades, various antibiotics have been added to acrylic bone cements, like gentamycin, tobramycin, erythromycin, cefuroxime, vancomycin, colistin, etc. [3,7,8]. The amount and type of the added antibiotic strongly depends on the treatment strategy, germs’ resistance, patient condition, etc. On the one hand, antibiotics are able to disturb the mechanical properties and even enhance the toxicity of bone cement [14], and on the other hand, bone cement heating during polymerization can interfere with the drugs’ antibacterial activity [15]. Nowadays there are no uniform standards for antibiotic dosage in bone cements. The ALBCs can be classified into two groups according to antibiotic concentration: low-dose ALBC and high-dose ALBC [2,5,16]. Most of the currently commercially available ALBC refer to the low-dose group since the drug mass fraction in them rarely exceeds 3 wt.% [7] and are basically employed in joint replacement surgery for infection prophylaxis [4,5]. However, occasionally low-dose ALBC cannot provide sufficient antibiotic elution to exceed the minimum-inhibitory concentrations of the germs [2,5]. Moreover, a comparatively low antibiotic concentration can induce the emergence of drug-resistant organisms [9,17]. Thus, the higher doses of antibiotics (antibiotic pairs) should be applied [7,9,18,19,20,21,22,23,24]. The typical antibiotic dosage in high-dose ALBC does not exceed 10 wt.% [7,18], whereas in particular cases, it can reach up to 20 wt.% [9,18]. Nevertheless, the widespread employment of high-dose ALBC (including the elaboration and implementation of the commercial products) is limited by cement preparation difficulties and mechanical issues. The large amount of crystalline antibiotics (e.g., bramycin and vancomycin) added to PMMA powder makes the further mixing with liquid MMA difficult [18]. Moreover, according to different reports, the mechanical stability of high-dose ALBC deteriorates when antibiotic content is close to 5 wt.% [3,16,25,26,27] or 10–15 wt.% [9,18,27,28,29,30,31]. Regardless the group of ALBC, the antibiotic powder is usually mixed with PMMA prior to the addition of liquid MMA. During and after the end of polymerization, the antibiotic particles are embedded in the polymer matrix; hence, the drug elution considerably depends on bone cement porosity [9,18]. The porosity in turn depends on many factors, like the particular conditions of the cement preparation and the form of antibiotic powder. The addition of the large antibiotic crystals can lead to greater bone cement porosity, and hence, an enhanced elution. Meanwhile, the incorporation of the large crystalline grains into polymer significantly effects the mechanical stability of ALBC [18]. Thus, the increasing drug elution while maintaining the mechanical stability of ALBC is highly required.

The micro- and nanoscale materials can be employed to increase the porosity/level of antibiotic elution from acrylic bone cements and to preserve the mechanical properties [5,32,33,34,35,36,37,38,39,40,41,42,43,44,45]. The influence on the ALBC drug release, antimicrobial properties, and mechanical properties was investigated after the incorporation of nanomaterials such as layered double hydroxide [36], silica mesoporous nanoparticles [33,38,39], carbon nanotubes (CNTs) [40,45], TiO_2_ nanotubes [5], and nanoclays [34]. The introduction of silica nanoparticles [42] and CNTs [40,45] preserved the mechanical properties of ALBC and led to a significant enhancement of the drug elution (e.g., 45% of the loaded gentamicin was released from the composite cement, while only 15% of the loaded gentamicin was released in the case of the ordinary ALBC [45]). CNTs can also improve cement adhesion [46], since the plain PMMA is bioinert, and hence, is not favorable for the adhesion, proliferation, and differentiation of osteoblasts [43]. Despite the promising results demonstrated by some synthetic nanoscale additives (e.g., CNTs), their widespread application is considerably limited due to the questionable cytotoxicity, difficulties in reproducibility in large-volume synthesis, as well as the high production cost. In this regard, a particular interest was drawn to completely biocompatible, low-cost, natural nanoscale materials (e.g., nanoclays, fibrillated cellulose, etc.) [34,47,48,49]. For instance, the halloysite nanotubes (HNTs) were used in composite ALBC (c-ALBC) [34]. The obtained c-ALBC possessed the enhanced total gentamicin elution (by 2–2.5 times), prolongated the drug release (over 300–400 h) and improved the adhesion and mechanical properties. The c-ALBC containing cellulose fibers revealed increased cumulative antibiotic elution (129% greater than for ALBC) with the simultaneous preservation of the mechanical properties [47], whereas in the work of S. Jacquart et al., the cellulose was successfully used as a carrier of nanosized silver [49]. Thus, modification of ALBC via the natural nanoscale additives seems to be the promising way in elaboration of the composite bone cement with an enhanced/prolongated drug release and improved mechanical properties.

In this work, the four composite bone cements containing vancomycin and 10 wt.% of the different natural additives were prepared. The employed additives are halloysite nanotubes (HNTs), micro-fibrillated cellulose (MFC), nano-fibrillated cellulose (NFC), and nano-crystalline cellulose (NCC). The effect of the additives incorporation on antibiotic elution was investigated as well as the microhardness of the obtained samples.

## 2. Materials and Methods

### 2.1. Raw Materials

In this research, commercially available surgical Simplex P radiopaque bone cement from Stryker Orthopedics (Limerick, Ireland) was used. The content of both components of bone cement is presented in Table S1, in the Supplementary Materials section. Carboxymethylcellulose sodium salt, sulfuric acid (H_2_SO_4_, 98%), and hydrogen peroxide (H_2_O_2_, 37 wt.%) HNTs and NCC were purchased from Sigma-Aldrich (St. Louis, MO, USA) and were further used without any treatment or purification. Vancomycin (PJSC “Krasfarma”, Krasnoyarsk, Russia) was chosen as one of the most effective and widely applied antimicrobial agents. Softwood sulfate bleached pulp was supplied by Arkhangelsk Pulp and Paper Mill (Arkhangelsk, Russia). Pulp characteristics are presented in Table S2, in the Supplementary Materials section.

### 2.2. MFC and NFC Preparation

The preparation procedures of fibrillated cellulose were previously described [50,51]. To obtain MFC, 2 g of carboxymethylcellulose sodium salt was added to 500 mL of the 5 wt.% water suspension of washed wood pulp. The obtained mixture was vigorously stirred for 1 h and then was passed through the Supermasscolloider Masuko MKCA6-5 (Kawaguchi, Japan). The produced microfibers were collected after a 2-day sedimentation of the treated solution.

To produce NFC, 2 g of the washed MFC were added to the solution obtained by mixing 0.8 g of H_2_O_2_, 16.9 g of concentrated sulfuric acid, and 10.4 g of distilled water. The final solution containing MFC (2 wt.%), H_2_O_2_ (1 wt.%), and H_2_SO_4_ (55 wt.%) was stirred in the Biosan ES20/40 orbital shaker incubator at 600 rpm for 4 h, filtrated, and washed. The obtained precipitate was diluted in distillated water to obtain a 3 wt.% suspension. NFC was produced via the treatment of the suspension using the Branson Digital Sonifier 450 (20 kHz, 400 W) (Brookfield, CT, USA) and the IKA ULTRA-TURRAX T-18 digital homogenizer (Staufen, Germany).

The morphology and length of the obtained cellulose fibrils were investigated with electron microscopy (Figure S1).

### 2.3. Bone Cement, ALBC and Composite ALBC Preparation

The pristine Simplex P bone cement was obtained according to the manufacturer protocol: 0.6 mL of the liquid (MMA) component was mixed with 1.2 g of the powder (PMMA) component. The obtained mixture was vigorously stirred for5 min and then placed in cubic form (1 cm × 1 cm × 1 cm). To avoid any undesired cavities in the sample volume, the bone cement was dried for 12 h under a weight of 1 kg. Then, the sample was retrieved from the form and dried at 50 °C in the Binder drying chamber for the next 12 h.

The preparation procedures of the ALBC and the composite ALBC (c-ALBC) are identical to those described above, with the exception of the addition of vancomycin and various micro/nano-sized fillers to PMMA powder. To obtain ALBC, well-ground vancomycin powder was added to the Simplex P powder component. The mass fraction of vancomycin in ALBC was 10 wt.% (regarding the mass of the powder component). The mixing of the two powders (2000 rpm using the Biosan Multi Speed Vortex MSV-3500 shaker (Riga, Latvia) for 20 min) was performed in one batch to ensure the homogeneous distribution of vancomycin in all ALBC samples. To obtain c-ALBC, different micro/nano-sized fillers were added to the initially mixed vancomycin and PMMA. Thus, the mass fraction of the filler in the composite ALBC was 13 wt.% (regarding the mass of the powder component). The obtained powder mixtures were blended again at 2000 rpm for 20 min in one batch to ensure the homogeneous distribution of fillers in the samples. A series of three samples were prepared for each composition (including BC and ALBC) to obtain the averaged results for the microhardness tests as well as the antibiotic elution study. The compositions of all prepared samples are collected in Table 1.

**Figure 1:**
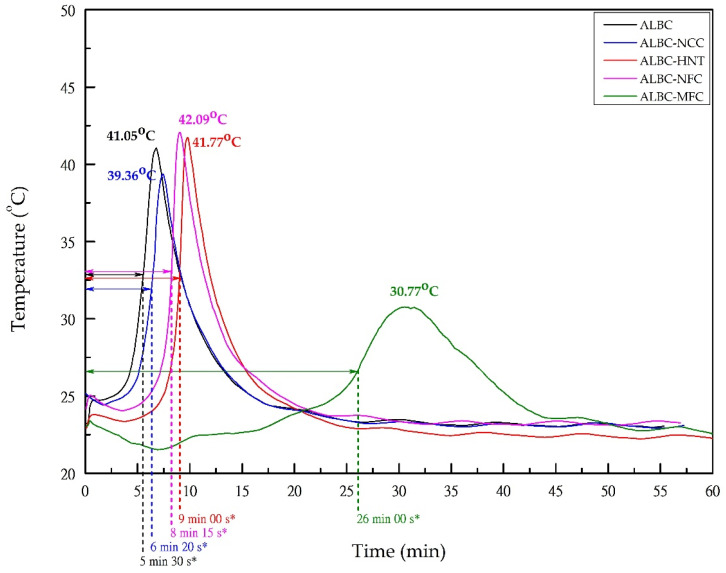
Temperature profiles and setting times of ALBC and c-ALBC samples. The corresponding setting times (denoted by double side arrows) and maximum temperatures reached during polymerization of the sample in the cubic form (1 cm × 1 cm × 1 cm) are presented in the Figure; *—the presented settings times are given from the start of temperature registration.

The exothermic process of MMA polymerization and setting time were characterized with the help of the Termex LTA-N digital lab thermometer (Tomsk, Russia). The thermometer head was introduced in the bone cement mass after 2 min of hand-mixing of the liquid and the corresponding powder components, and the provided registration of the temperature occurred every 5 s for 1 h. All measurements were performed at the temperature of 23 °C. The corresponding temperature profiles, including the maximum temperatures reached during polymerization of each sample, are collected in Figure 1. The same figure contains the information about the setting time of each sample. The setting time was defined as the time from the onset of the ALBC components’ mixing until the surface temperature reaches ½ of the maximum temperature [52,53,54]. It should be noted that the setting times presented in Figure 1 were measured from the beginning of temperature registration but not from the moment of the ALBC components’ mixing; thus, to obtain the real setting times, the values presented in the Figure 1 should be extended by 2 min.

Then, the obtained samples were retrieved from the forms and weighted. In order to estimate the effect of the additive incorporation on the degree of polymerization, the concentration of MMA released during the 10 min extraction in 4 mL of n-hexane was measured with the help of a gas chromatograph equipped with the flame ionization detector Chromatek-Crystal 5000.2 (ZAO SKB Chromatek, Yoshkar-Ola, Russia) and the HP-5 ms column (25 m, 0.25 mm). Quantitative analysis was performed using a calibration graph according to the method of external standard.

### 2.4. Antibiotic Elution Study

To study vancomycin elution from the ALBC and c-ALBC samples, the corresponding protocols/methods described previously were employed [55,56,57,58]. The bone cement samples placed in 10 mL vials with distillated water were stirred at 150 rpm with the Biosan environmental shaker-incubator ES-20/60. After regular time intervals, 10 mL of the solution was poured off and a new/fresh portion of distilled water (10 mL) was added to the vial. The concentration of the antibiotic released from the ALBC and c-ALBC samples was determined by measuring the optical density of the corresponding aqueous solution (vancomycin absorbance peak at 280 nm) in a 200–800 nm range using a Cary 60 UV-Vis spectrophotometer from Agilent Technologies (Santa Clara, CA, USA) and the corresponding calibration curve (see Figure S2). To obtain the total concentration eluted after *n* measurements, the concentrations measured in each of the *n* measurements should be summed up. The first 10 measurements of the solution’s optical density were taken every hour as the antibiotic is most actively eluted during the first hours. The next two measurements were taken after 24 h, followed by two measurements after 48 h and the last measurement after 115 h.

It should be noted that the control experiment was carried out with a sample of bone cement to ensure that the optical density of the solution did not increase over time at 280 nm.

### 2.5. Microhardness Tests

Microhardness measurements of bone cement, ALBC, and c-ALBC were performed using a Fischerscope HM2000 S nanoindenter after the antibiotic elution study. The shape and size of the samples were the same as in the elution study. During the measurements, the load and displacement of the indenter were registered. Every sample of the three-cube series was subjected to 10 loads in a 0–300 mN range, where the maximum force of 300 mN was reached in an application time of 20 s, and the creep at maximum force was 10 s. Martens hardness (HM) was calculated based on the load/displacement relationships.

### 2.6. Electron Microscopy

The distribution of vancomycin grains and additives in the PMMA matrix as well as the MFC shape and length were investigated with the help of scanning electron microscopy (SEM). The samples retrieved after the elution study and microhardness test were crashed in the mortar and covered by a 10–15 nm gold layer via magnetron sputtering using Quorum Technologies Q150R Plus sputter coater. The JEOL JIB 4501 multibeam system (Akishima, Tokyo) was used, and the SEM micrographs were acquired in BSE mode at an accelerating voltage of 10 kV.

To study the form and structure of the employed HNT, NCC and NFC transmission electron microscopy (TEM) was employed. The corresponding micrographs were acquired using the JEOL JEM 2100 UHR microscope in TEM brightfield mode at an accelerating voltage of 200 kV. To enhance the contrast of cellulose fibrils with low electron-scattering power, the negative staining (using a 2% solution of phosphotungstic acid, pH = 7.0) was applied.

### 2.7. Statistics

The data collated in all experiments were evaluated for statistical significance using a one-way analysis of variance with *p* < 0.05 denoting the significance. Post hoc tests were conducted using the Student–Newman–Keuls method. All the tests were conducted using commercially available software (SAS version 8.02; SAS Institute, Cary, NC, USA).

## 3. Results and Discussion

To explore the influence of the different fillers on the antibiotic elution, a three-cube series of c-ALBC containing 10 wt.% of HNT, NCC, NFC, and MFC were prepared. To study the effect of the fillers’ incorporation in Simplex P bone cement, the key parameters, such as the maximum polymerization temperature, setting time, and MMA leaching degree, were evaluated. Since bone cement curing is a complex process, the introduction of the additives may significantly affect MMA polymerization, and hence, the polymerization temperature, setting time, and the degree of polymerization. According to the previous reports, various types of additives can either reduce [59] or prolongate [54,60] the setting time; in some cases, the setting time is not changed [48,61,62]. Application of different additives can also vary the temperature of polymerization. Thus, the addition of materials such as bioactive glass [62], chitosan [48], cellulose [48], MWCNTs [54], and hydroxyapatite/chitosan [60] resulted in a decrease in the maximum temperature of polymerization. At the same time incorporation of Mg [48] or AgNPs [63] led to raise of the temperature of polymerization. The latter is highly undesired because of inflammation, protein denaturation, and bone tissue necrosis [52,54].

In the present work, almost no effect on PMMA polymerization was observed in the case of HNT-, NCC-, and NFC-containing c-ALBCs. According to the results presented in Figure 1, the addition of NCC, HNT, and NFC led to little increase in the setting time and an insignificant variation in the temperature of polymerization (Table 2). However, a strong smell of MMA was observed even after 12 h of drying at room temperature in the case of the MFC-containing samples. Despite the considerable reduction in the polymerization exothermic effect (the maximum temperature of polymerization does not exceed 30.8 °C), MFC incorporation increased the setting time almost by four times, indicating the decrease in the PMMA polymerization rate (Figure 1).

A mechanism of the additive influence on the polymerization temperature is not always clear and can vary in the case of different materials. Thus, for example, MWCNTs have very high thermal conductivity and can act as “heat sinks” within the polymer matrix [54]. Moreover, the carboxyl group of MMA can link with hydroxyl groups via hydrogen bonding [64], thus decelerating the polymerization reaction. Nanoscale HNT, NCC, and NFC possess neither good thermal conductivity nor a great number of hydroxyl groups on its surface, which presumably resulted in a negligible effect on the PMMA polymerization rate. Unlike powder-like HNT, NCC, and NFC, MFC has comparatively long fibrils containing a great number of OH-groups. When mixed with MMA, the MFC fibrils might adsorb monomer molecules (linking them via hydrogen bonds), which eventually affects the rate of the polymerization reaction and its conversion. The maximum temperature observed for MFC-containing ALBC in present research is close to the value observed previously [48]. Nevertheless, it should be underlined that the direct comparison of the polymerization temperatures from different works might be ambiguous since this parameter also strongly depends on the size/shape of the BC sample, the ratio of monomer to polymer, the conditions of the temperature registration (e.g., in vitro or in vivo studies), as well as the degree of heat waste [52,65,66].

The polymerization methods mentioned above also strongly affects the degree of polymerization, and thus, the amount of unreacted monomer [67]. A high concentration of MMA, which can leach into the adjacent tissues, may cause irritation, inflammation, and chemical necrosis [52,67]. It is noted that MMA can also participate in the triggering of bone cement implantation syndrome, which is a lethal syndrome with complex physiological changes after bone cement introduction [68].

**Figure 2:**
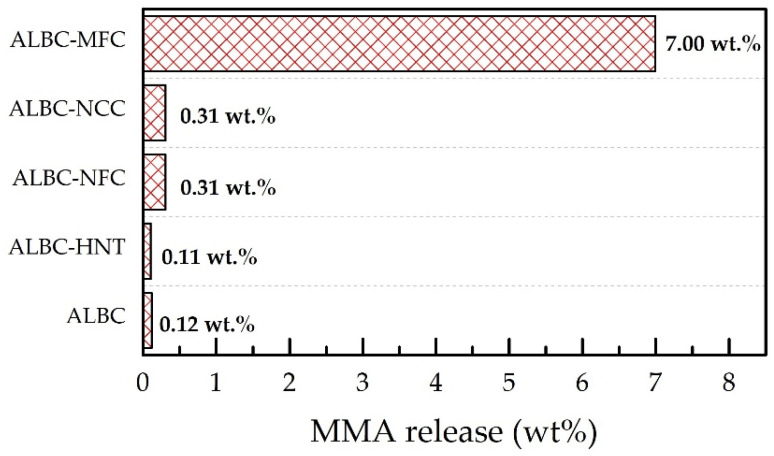
MMA leaching after 10 min extraction in 4 mL of n-hexane. The corresponding released MMA quantities (in regard to the total weight of sample) are presented in the figure.

The results of the MMA leach investigation are presented in Figure 2: 7 wt.% of MMA were not polymerized during the first hour in the MFC-containing sample, whereas the corresponding values of ALBC and the HNT-, NCC-, and NFC-containing c-ALBC samples are 0.12 wt.%, 0.11 wt.%, 0.31 wt.%, and 0.31 wt.%, respectively. Besides the fact that MFC fibrils could absorb/bind monomer molecules (e.g., via hydrogen bonding), and thus, retard MMA polymerization, it might be assumed that high MMA leaching can be caused by the highest porosity of the MFC-containing sample compared to other c-ALBCs. It should be noted that a mixing of PMMA powder with MFC as well as the manipulation with the substance after the addition of the liquid part was significantly complicated because of the fibrous nature of cellulose. Summing up, despite the considerable reduction in the maximum temperature of MMA polymerization, one can conclude that MFC is not a suitable filler for ALBC modification in real surgical application.

**Figure 3:**
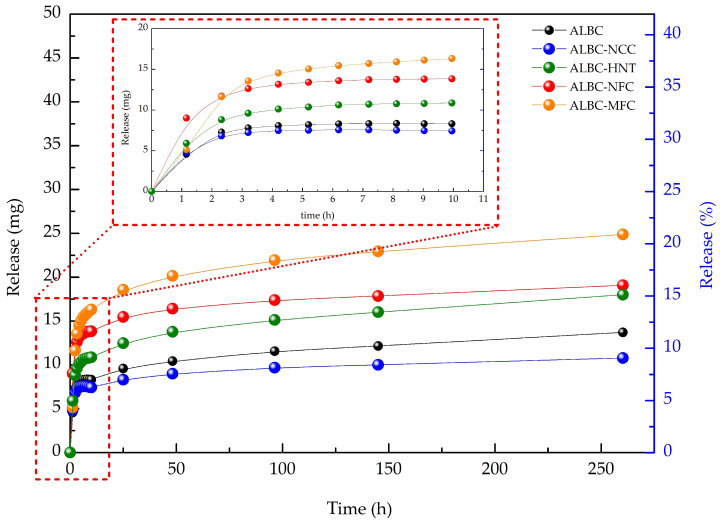
Vancomycin elution from ALBC and c-ALBC over 269 h. The insert: vancomycin elution during first 10 h. For visual convenience, the data are presented in scatter-line format.

The antibiotic elution curves of ALBC and c-ALBC are presented in Figure 3, according to which c-ALBC demonstrated a higher vancomycin release compared to ALBC with the exception of the NCC-containing samples. The amounts of vancomycin released per hour by the ALBC and c-ALBC samples are presented in Table 3. Among c-ALBC, the antibiotic release decreases in the row MFC > NFC ≥ HNT > NCC. As it follows from Figure 3 and Table 3, the vancomycin elution in all samples is not constant: the phase of exponential increase in drug release during the first ten hours is followed by the declining phase and plateau in a few days. According to the literature data, such a way of vancomycin elution is typical for PMMA-based ALBC [4]. Nevertheless, despite the same shape of the vancomycin elution curves of ALBC (for instance, see [27]), the kinetics and quantity of the released drug can vary in different studies. After 260 h of the elution study, 11.4% of the loaded vancomycin was released. This value exceeds the antibiotic release values observed in the previous studies for various antibiotics [5,27,40,42,58]; however, it is lower than some values observed in other studies [23,69,70]. The observed differences of the amount of the released antibiotic and its elution rate are not surprising, since the burst diffusion of the first hours depends on such factors such as the shape and dimensions of the sample/spacer (hence, the diffusion area), porosity, and the hydrophilicity of the surface [4]. Moreover, elution significantly depends on the antibiotic blending procedure [22].

In addition to what was mentioned above, the interpretation of antibiotic elution in c-ALBC is more complicated since the introduced fillers can act as porogens and additional paths for water/drug diffusion. Taking this fact into account, as well as the great variety of the fillers [5,33,34,35,36,38,40,42,45], the direct comparison of antibiotic elution reported in different studies is complicated. According to the literature review, there is only one study devoted to the application of HNT loaded with gentamicin as an additive in c-ALBC [34]. After 250 h of elution in deionized water, the c-ALBC containing 15 wt.% of halloysite released 60% of the total antibiotic amount, whereas the incorporation of 13 wt.% of halloysite in the present study led to a 15% release of vancomycin. In addition, some reports are devoted to the application of cellulose fibrils in acrylic bone cements as carries of silver nanoparticles, which also enhances the mechanical strength of composite material [48,49]. To the best of our knowledge, there is only one study devoted to the application of this natural biopolymer in c-ALBCs to improve antibiotic elution [47]. In the 35th day of the elution study, the proportion of the released drug to the loaded drug was 19.1%, which is close to the corresponding values observed in the present study (15.9% for NFC and 20.7% for MFC). However, the incorporation of carbon nanotubes (CNTs) [40,45], TiO_2_ nanotubes (TNTs) [5], mesoporous silica nanoparticles (MSN) [40,42,71], and hydroxyapatite particles (HAP) [40] leads to the enhancement of antibiotic elution, which is more considerable than it was observed in this work.

The antibiotic elution study was followed by the microhardness tests. Our study has some limitations. The investigation of microhardness allows us to probe the mechanical properties of the thin surface layer of ALBC/c-ALBC but not the bulk of the sample. It should also be underlined that microhardness tests were not performed prior to the elution study. A big number of indentations made on the surface of each sample and the induced cracks might have an unpredictable effect on vancomycin release in the elution study.

**Figure 4:**
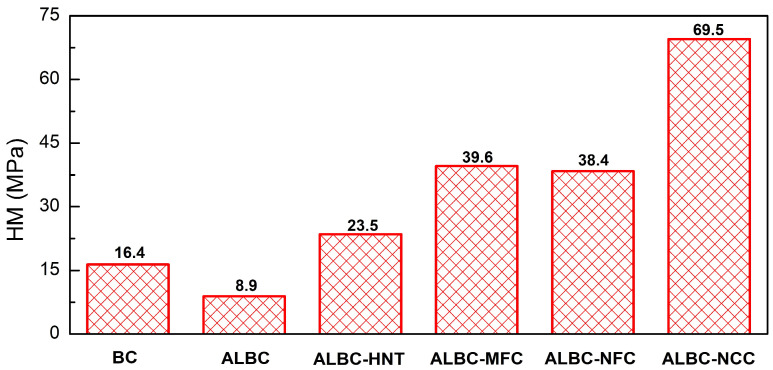
Microhardness tests of the samples of the bone cement, ALBC, and c-ALBC. The corresponding values of Martens hardness (HM) are indicated in the figure.

As it follows from Figure 4, the addition of 10 wt.% vancomycin to the polymer matrix considerably reduced the microhardness of ALBC compared to pristine bone cement. According to the previous reports, the introduction of antibiotics compromises the mechanical properties of bone cement [25,26,27,31,72,73,74,75,76,77,78,79]. Many factors such as antibiotic weight content [25,26,27,28,73,75,78], antibiotic (or antibiotics) type [31,74,77,79], and mixing procedure (e.g., hand- or vacuum-mixing) [56,80,81] have different effects on ALBC’s mechanical properties. During antibiotic elution (bone cement aging), a number of voids/pores are formed, reducing ALBC’s mechanical stability [26,73,77]. Obviously, the bigger mass content of drug results in a greater porosity of the polymer matrix [28]. It is worth noting that porosity can be controlled not only via antibiotic content but also via ALBC’s preparation conditions. Thus, for instance, the preparation of ALBC under a vacuum can reduce the number of air voids and cracks in PMMA [56,80,81]. In addition, various antibiotic molecules can react with MMA, impending the polymerization process, and thus, weaking the ALBC. Moreover, the ALBC failure might begin from the antibiotic agglomerations in the polymer matrix [27].

The introduction of the fillers led to a significant increase in c-ALBC microhardness (Figure 4). It should be underlined that the incorporation of HNTs [34] and cellulose [47] in PMMA led to a considerable improvement of the mechanical properties of the bone cements previously. The samples containing NCC revealed the highest microhardness value. This result is not surprising because among all the used additives, NCC possesses the outstanding mechanical properties (elastic modulus of 110–220 GPa, Young’s modulus of 150 GPa, and tensile strength of 10 GPa [82]). Thus, one can conclude that the ALBC modification with different additives can significantly improve the mechanical properties and counteract the negative effect of the antibiotic loading.

**Figure 5:**
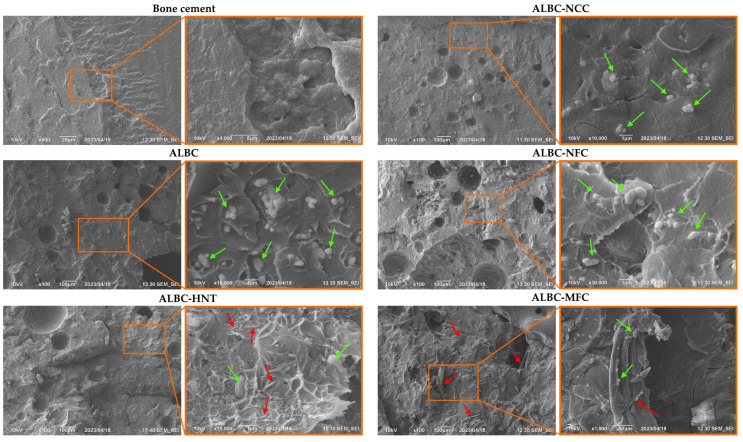
SEM micrographs of bone cement, ALBC, and c-ALBC. The orange rectangular indicates the acquisition zone of the micrographs of the higher magnification; the red and green arrows indicate the fillers and the antibiotic microgranules, respectively.

The microstructure of the BC, ALBC, and composite ALBC was investigated at the fracture surface of the cubic samples employing SEM (Figure 5). According to the low magnification SEM micrographs, the number of pores in the PMMA matrix increases significantly when the antibiotic and fillers are introduced into its composition. SEM micrographs of the ALBC and c-ALBC samples at higher magnifications exhibit micrograins of the antibiotic (marked by green arrows in the figure), as well as in some cases, the additive particles/fibrils (marked by red arrows in the figure).

Obviously, the antibiotic release happens at the sample’s surface and from deep pores/cracks in the polymer. Assuming that the antibiotic powder is homogeneously dispersed in the PMMA, most of the drug remains inaccessible for diffusion/release. The significantly increased release of vancomycin in the c-ALBC samples is associated with the increased porosity. The introduction of the additive enhances the permeability to water/drug elution via two ways: (1) the additives influence the polymerization and setting of PMMA, resulting in a higher porosity level of the final composite (see Figure 5); and (2) the additives act as additional conducting channels through which water penetrates inside the polymer matrix, and the dissolved antibiotic diffuses outward. For instance, according to R. Mori et al., cellulose fibrils act as hydrophilic pathways in the hydrophobic polymer matrix, ensuring the better antibiotic diffusion. Thus, the effectiveness of antibiotic release can depend not only on the proximity of antibiotic microgranules and the additive, but also on the particle length of the latter.

Table 4 summarizes the amount of vancomycin released (in % of the weight of the loaded antibiotic) from the ALBC and c-ALBC samples, as well as the average particle lengths of the additives used in the present work.

As shown in Table 4, the amount of the released antibiotic is in a good agreement with the additive particle length: the longer the particle length of the additive, the more antibiotic is released. This observation forms the basis of our hypothesis: the modifying additives create a kind of channel system within the polymer matrix. Obviously, the longer the additive particle/fibril, the more likely it crosses with another one, as well as with antibiotic microgranules.

The proposed hypothesis makes it possible to explain the comparatively low vales of vancomycin elution for the NCC-containing c-ALBC sample. NCC has the shortest fibril length among all analyzed additives (see Table 4). Thus, NCC particles as well as vancomycin microgranules are likely isolated from each other and do not form a network within the polymer matrix. Moreover, NCC probably hinders the diffusion of the antibiotic through the natural pores/channels/cracks of the bone cement, resulting in lower values of antibiotic release compared to non-modified ALBC.

Summing up, based on the results of the vancomycin elution and the mechanical tests, as well as the specifics of c-ALBC preparation, we conclude that NFC is the most promising modifying additive. The application of highly biocompatible and low-cost cellulose nanofibrils provides both an enhanced antibiotic release and mechanical stability of c-ALBC, which opens up real prospects for the application of the latter in medical practice.

## 4. Conclusions

In this work, we considered four types of natural materials with different morphologies (different shapes and sizes: nanotubes, fibers, and particles) as potential additives to commercial PMMA bone cement preloaded with vancomycin. The application of nanoscale HNT, NFC, and NCC did not considerably affect PMMA polymerization (setting time and maximum temperature of polymerization). Nevertheless, the addition of MFC to ALBC led to a significant setting time extension and a polymerization temperature drop, indicating a significant reduction in the polymerization rate. This observation is supported by the investigations of MMA leaching from the bone cements: the level of monomer release for ALBC and the HNT-, NCC-, NFC-containing c-ALBCs do not exceed 0.5 wt.%; meanwhile, the same value for the MFC-containing sample is 7 wt.%. Summing up, the low polymerization reaction rate, high level of MMA leaching (which can cause serious health issues if the corresponding c-ALBC applied), and the difficulties associated with bone cement preparation make MFC an unsuitable additive for real surgical application.

The introduction of the additives under investigation resulted in a significant improvement of its microhardness, even though the initial addition of the antibiotic to bone cement led to a decrease in this value. Composite ALBC demonstrated an enhanced vancomycin release compared to ALBC, with the exception of the NCC-containing samples. The drug release from the composite cement might be affected by the additive particle length, which is likely due to the network formation of the added particles within the polymer matrix. NCC particles having the smallest size among the tested additives are likely to become disconnected from each other within PMMA, which hinder the diffusion of the antibiotic through the natural pores/channels/cracks of the bone cement; meanwhile, the fibrous (MFC, NFC) and tubular (halloysite) additives promote antibiotic diffusion. Thus, the drug elution properties of the composite bone cements can be tuned by using additives with a suitable morphology (particle length).

Based on the results of c-ALBC polymerization (setting time and MMA leaching), the analysis of mechanical properties, the amount of released antibiotic, and the specifics of the composite cements’ preparation, we consider NFC to be the most promising additive to modify bone cement, taking into account that the cellulose is abundant, and hence, a low-cost, ecofriendly, and biocompatible material.

## Figures and Tables

**Table 1:** The compositions of Simplex P bone cement, ALBC, and composite ALBC samples.

Sample	PMMA, g	MMA, mL	Vancomycin		Filler		
			Mass, g	Content *, wt.%	Name	Mass, g	Content *, wt.%
BC	1.2	0.6	0	0	—	—	0
ALBC	1.08	0.6	0.12	10	—	—	0
ALBC-HNT	1.08	0.6	0.12	9	HNT	0.18	13
ALBC-NCC	1.08	0.6	0.12	9	NCC	0.18	13
ALBC-NFC	1.08	0.6	0.12	9	NFC	0.18	13
ALBC-MFC	1.08	0.6	0.12	9	MFC	0.18	13

* the content is given regarding the powder component.

**Table 2:** The setting time and maximum temperature of polymerization of ALBC and c-ALBC samples.

Sample		Setting Time, mm:ss	Maximum Temperature, °C
ALBC		07:30	41.1
c-ALBC	10 wt.% NCC	08:00	39.4
	10 wt.% HNT	10:15	41.8
	10 wt.% NFC	11:00	42.1
	10 wt.% MFC	28:00	30.8

**Table 3:** The rate of vancomycin released from ALBC and c-ALBC. The values are in brackets—SD, *n* = 3.

Time (h)	ALBC (mg/h)	c-ALBC (mg/h)			
		NFC	NCC	MFC	HNT
1	40.55(0.12)	90.02(0.18)	40.84(0.08)	50.15(0.11)	50.92(0.17)
2	30.64(0.07)	50.85(0.02)	30.41(0.09)	50.82(0.06)	40.41(0.11)
3	20.60(0.01)	40.21(0.05)	20.41(0.01)	40.52(0.03)	30.20(0.02)
4	20.02(0.02)	30.29(0.04)	10.87(00)	30.64(0.03)	20.53(0.01)
5	10.64(0.01)	20.68(0.02)	10.50(00)	30.01(0.01)	20.07(0.00)
6	10.38(0.01)	20.27(0.02)	10.26(00)	20.58(0.01)	10.77(0.00)
7	10.19(0.00)	10.96(0.01)	10.08(00)	20.25(0.01)	10.53(0.00)
8	10.04(0.01)	10.72(0.01)	00.94(00)	10.99(0.01)	10.35(0.00)
9	00.92(0.00)	10.53(0.01)	00.83(00)	10.79(0.01)	10.20(0.01)
10	00.83(0.01)	10.38(0.01)	00.74(00)	10.63(0.00)	10.09(0.01)
25	00.40(0.05)	00.64(0.07)	00.35(0.02)	00.77(0.04)	00.52(03)
48	00.22(0.01)	00.34(0.03)	00.19(0.03)	00.42(0.02)	00.29(02)
96	00.12(0.02)	00.18(0.00)	00.10(0.03)	00.23(0.01)	00.16(0.01)
145	00.08(0.00)	00.12(0.00)	00.07(0.01)	00.16(0.00)	00.11(0.02)
260	00.05(0.01)	00.07(0.01)	00.04(0.01)	00.10(0.01)	00.07(0.03)

**Table 4:** The amount of the released vancomycin from ALBC and c-ALBC samples and the average length of the additive particle/fibril.

Sample	Vancomycin Released (% *)	Length of Additive Particle (μm)
ALBC	11.4	—
ALBC-HNT	15.0	0.2–2 [83]
ALBC-NCC	9.0	0.1–0.3 [82]
ALBC-NFC	15.9	0.2–3 **
ALBC-MFC	20.7	30–70 **

* The amount of the released vancomycin (in %) is given in regard to its theoretical content, see Table 1. ** The measurements of MFC and NFC fibril lengths were performed in this study.

## Data Availability

Not applicable.

## Supplementary Materials

The following supporting information can be downloaded at: https://www.mdpi.com/article/10.3390/polym15183757/s1, Figure S1: Electron microscopy micrographs of HNT (a), NCC (b), MFC (c), and NFC (d); Figure S2: Calibration curve for determination of vancomycin concentration in aqueous solution by its optical density; Table S1: Composition of surgical Simplex P radiopaque bone cement; Table S2: Pulp characteristics.

## References

[1] Bourne R.B. (2004). Prophylactic Use of Antibiotic Bone Cement An Emerging Standard—In the Affirmative. J. Arthroplast..

[2] Jiranek W.A., Hanssen A.D., Greenwald A.S. (2006). Antibiotic-Loaded Bone Cement for Infection Prophylaxis in Total Joint Replacement. J. Bone Jt. Surg..

[3] Vaishya R., Chauhan M., Vaish A. (2013). Bone Cement. J. Clin. Orthop. Trauma.

[4] James A., Larson T. (2015). Acute Renal Failure after High-Dose Antibiotic Bone Cement: Case Report and Review of the Literature. Ren. Fail..

[5] Shen S.-C., Letchmanan K., Chow P.S., Tan R.B.H. (2019). Antibiotic Elution and Mechanical Property of TiO_2_ Nanotubes Functionalized PMMA-Based Bone Cements. J. Mech. Behav. Biomed. Mater..

[6] Dong T., Huang Q., Sun Z. (2023). Antibiotic-Laden Bone Cement for Diabetic Foot Infected Wounds: A Systematic Review and Meta-Analysis. Front. Endocrinol..

[7] Hollyer I., Ivanov D., Kappagoda S., Lowenberg D.W., Goodman S.B., Amanatullah D.F. (2023). Selecting a High-dose Antibiotic-laden Cement Knee Spacer. J. Orthop. Res..

[8] Steadman W., Chapman P.R., Schuetz M., Schmutz B., Trampuz A., Tetsworth K. (2023). Local Antibiotic Delivery Options in Prosthetic Joint Infection. Antibiotics.

[9] Wang L., Lu S., Luo W., Wang G., Zhu Z., Liu Y., Gao H., Fu C., Ren J., Zhang Y. (2023). Efficacy Comparison of Antibiotic Bone Cement–Coated Implants and External Fixations for Treating Infected Bone Defects. Int. Orthop. (SICOT).

[10] Block J.E., Stubbs H.A. (2005). Reducing the Risk of Deep Wound Infection in Primary Joint Arthroplasty with Antibiotic Bone Cement. Orthopedics.

[11] Randelli P., Evola F.R., Cabitza P., Polli L., Denti M., Vaienti L. (2010). Prophylactic Use of Antibiotic-Loaded Bone Cement in Primary Total Knee Replacement. Knee Surg Sports Traumatol. Arthrosc..

[12] Nowinski R.J., Gillespie R.J., Shishani Y., Cohen B., Walch G., Gobezie R. (2012). Antibiotic-Loaded Bone Cement Reduces Deep Infection Rates for Primary Reverse Total Shoulder Arthroplasty: A Retrospective, Cohort Study of 501 Shoulders. J. Shoulder Elb. Surg..

[13] Masri B.A., Duncan C.P., Beauchamp C.P. (1998). Long-Term Elution of Antibiotics from Bone-Cement. J. Arthroplast..

[14] Funk G.A., Menuey E.M., Cole K.A., Schuman T.P., Kilway K.V., McIff T.E. (2019). Radical Scavenging of Poly(Methyl Methacrylate) Bone Cement by Rifampin and Clinically Relevant Properties of the Rifampin-Loaded Cement. Bone Jt. Res..

[15] Levack A.E., Turajane K., Yang X., Miller A.O., Carli A.V., Bostrom M.P., Wellman D.S. (2021). Thermal Stability and in Vitro Elution Kinetics of Alternative Antibiotics in Polymethylmethacrylate (PMMA) Bone Cement. J. Bone Jt. Surg..

[16] Hanssen A.D. (2004). Prophylactic Use of Antibiotic Bone Cement An Emerging Standard—In Opposition. J. Arthroplast..

[17] Belt H.V.D., Neut D., Schenk W., Horn J.R.V., Mei H.C.V.D., Busscher H.J. (2001). Infection of Orthopedic Implants and the Use of Antibiotic-Loaded Bone Cements: A Review. Acta Orthop. Scand..

[18] Hanssen A.D., Spangehl M.J. (2004). Practical Applications of Antibiotic-Loaded Bone Cement for Treatment of Infected Joint Replacements. Clin. Orthop. Relat. Res..

[19] Hsieh P.-H., Chen L.-H., Chen C.-H., Lee M.S., Yang W.-E., Shih C.-H. (2004). Two-Stage Revision Hip Arthroplasty for Infection with a Custom-Made, Antibiotic-Loaded, Cement Prosthesis as an Interim Spacer. J. Trauma Inj. Infect. Crit. Care.

[20] Cui Q., Mihalko W.M., Shields J.S., Ries M., Saleh K.J. (2007). Antibiotic-Impregnated Cement Spacers for the Treatment of Infection Associated with Total Hip or Knee Arthroplasty. J. Bone Jt. Surg..

[21] Luu A., Syed F., Raman G., Bhalla A., Muldoon E., Hadley S., Smith E., Rao M. (2013). Two-Stage Arthroplasty for Prosthetic Joint Infection. J. Arthroplast..

[22] Amin T.J., Lamping J.W., Hendricks K.J., McIff T.E. (2012). Increasing the Elution of Vancomycin from High-Dose Antibiotic-Loaded Bone Cement: A Novel Preparation Technique. J. Bone Jt. Surg..

[23] Gálvez-López R., Peña-Monje A., Antelo-Lorenzo R., Guardia-Olmedo J., Moliz J., Hernández-Quero J., Parra-Ruiz J. (2014). Elution Kinetics, Antimicrobial Activity, and Mechanical Properties of 11 Different Antibiotic Loaded Acrylic Bone Cement. Diagn. Microbiol. Infect. Dis..

[24] Stevens C.M., Tetsworth K.D., Calhoun J.H., Mader J.T. (2005). An Articulated Antibiotic Spacer Used for Infected Total Knee Arthroplasty: A Comparative in Vitro Elution Study of Simplex^®^ and Palacos^®^ Bone Cements. J. Orthop. Res..

[25] Dunne N., Hill J., Mcafee P., Todd K., Kirkpatrick R., Tunney M., Patrick S. (2007). In Vitro Study of the Efficacy of Acrylic Bone Cement Loaded with Supplementary Amounts of Gentamicin: Effect on Mechanical Properties, Antibiotic Release, and Biofilm Formation. Acta Orthop..

[26] Pelletier M.H., Malisano L., Smitham P.J., Okamoto K., Walsh W.R. (2009). The Compressive Properties of Bone Cements Containing Large Doses of Antibiotics. J. Arthroplast..

[27] Lee S.-H., Tai C.-L., Chen S.-Y., Chang C.-H., Chang Y.-H., Hsieh P.-H. (2016). Elution and Mechanical Strength of Vancomycin-Loaded Bone Cement: In Vitro Study of the Influence of Brand Combination. PLoS ONE.

[28] He Y., Trotignon J.P., Loty B., Tcharkhtchi A., Verdu J. (2002). Effect of Antibiotics on the Properties of Poly(Methylmethacrylate)-Based Bone Cement. J. Biomed. Mater. Res..

[29] Bishop A.R., Kim S., Squire M.W., Rose W.E., Ploeg H.-L. (2018). Vancomycin Elution, Activity and Impact on Mechanical Properties When Added to Orthopedic Bone Cement. J. Mech. Behav. Biomed. Mater..

[30] Wall V., Nguyen T.-H., Nguyen N., Tran P.A. (2021). Controlling Antibiotic Release from Polymethylmethacrylate Bone Cement. Biomedicines.

[31] Paz E., Sanz-Ruiz P., Abenojar J., Vaquero-Martín J., Forriol F., Del Real J.C. (2015). Evaluation of Elution and Mechanical Properties of High-Dose Antibiotic-Loaded Bone Cement: Comparative “In Vitro” Study of the Influence of Vancomycin and Cefazolin. J. Arthroplast..

[32] Liu-Snyder P., Webster T. (2008). Developing a New Generation of Bone Cements with Nanotechnology. CNANO.

[33] Shen S.-C., Ng W.K., Shi Z., Chia L., Neoh K.G., Tan R.B.H. (2011). Mesoporous Silica Nanoparticle-Functionalized Poly(Methyl Methacrylate)-Based Bone Cement for Effective Antibiotics Delivery. J. Mater. Sci. Mater. Med..

[34] Wei W., Abdullayev E., Hollister A., Mills D., Lvov Y.M. (2012). Clay Nanotube/Poly(Methyl Methacrylate) Bone Cement Composites with Sustained Antibiotic Release. Macromol. Mater. Eng..

[35] Arora M. (2013). Polymethylmethacrylate Bone Cements and Additives: A Review of the Literature. WJO.

[36] Kapusetti G., Mishra R.R., Srivastava S., Misra N., Singh V., Roy P., Singh S.K., Chakraborty C., Malik S., Maiti P. (2013). Layered Double Hydroxide Induced Advancement in Joint Prosthesis Using Bone Cement: The Effect of Metal Substitution. J. Mater. Chem. B.

[37] No Y.J., Roohani-Esfahani S., Zreiqat H. (2014). Nanomaterials: The next Step in Injectable Bone Cements. Nanomedicine.

[38] Slane J., Vivanco J., Ebenstein D., Squire M., Ploeg H.-L. (2014). Multiscale Characterization of Acrylic Bone Cement Modified with Functionalized Mesoporous Silica Nanoparticles. J. Mech. Behav. Biomed. Mater..

[39] Slane J., Vivanco J., Meyer J., Ploeg H.-L., Squire M. (2014). Modification of Acrylic Bone Cement with Mesoporous Silica Nanoparticles: Effects on Mechanical, Fatigue and Absorption Properties. J. Mech. Behav. Biomed. Mater..

[40] Shen S.-C., Ng W.K., Dong Y.-C., Ng J., Tan R.B.H. (2016). Nanostructured Material Formulated Acrylic Bone Cements with Enhanced Drug Release. Mater. Sci. Eng. C.

[41] Lewis G. (2017). Properties of Nanofiller-Loaded Poly (Methyl Methacrylate) Bone Cement Composites for Orthopedic Applications: A Review: PROPERTIES OF NANOFILLER-LOADED BONE CEMENTS. J. Biomed. Mater. Res..

[42] Letchmanan K., Shen S.-C., Ng W.K., Kingshuk P., Shi Z., Wang W., Tan R.B.H. (2017). Mechanical Properties and Antibiotic Release Characteristics of Poly(Methyl Methacrylate)-Based Bone Cement Formulated with Mesoporous Silica Nanoparticles. J. Mech. Behav. Biomed. Mater..

[43] Sa Y., Yang F., Wang Y., Wolke J.G.C., Jansen J.A., Chun H.J., Park C.H., Kwon I.K., Khang G. (2018). Modifications of Poly(Methyl Methacrylate) Cement for Application in Orthopedic Surgery. Cutting-Edge Enabling Technologies for Regenerative Medicine.

[44] Bistolfi A., Ferracini R., Albanese C., Vernè E., Miola M. (2019). PMMA-Based Bone Cements and the Problem of Joint Arthroplasty Infections: Status and New Perspectives. Materials.

[45] Al Thaher Y., Khalil R., Abdelghany S., Salem M.S. (2022). Antimicrobial PMMA Bone Cement Containing Long Releasing Multi-Walled Carbon Nanotubes. Nanomaterials.

[46] Wang C., Yu B., Fan Y., Ormsby R.W., McCarthy H.O., Dunne N., Li X. (2019). Incorporation of Multi-Walled Carbon Nanotubes to PMMA Bone Cement Improves Cytocompatibility and Osseointegration. Mater. Sci. Eng. C.

[47] Mori R., Nakai T., Enomoto K., Uchio Y., Yoshino K. (2011). Increased Antibiotic Release from a Bone Cement Containing Bacterial Cellulose. Clin. Orthop. Relat. Res..

[48] Wekwejt M., Michalska-Sionkowska M., Bartmański M., Nadolska M., Łukowicz K., Pałubicka A., Osyczka A.M., Zieliński A. (2020). Influence of Several Biodegradable Components Added to Pure and Nanosilver-Doped PMMA Bone Cements on Its Biological and Mechanical Properties. Mater. Sci. Eng. C.

[49] Jacquart S., Girod-Fullana S., Brouillet F., Pigasse C., Siadous R., Fatnassi M., Grimoud J., Rey C., Roques C., Combes C. (2022). Injectable Bone Cement Containing Carboxymethyl Cellulose Microparticles as a Silver Delivery System Able to Reduce Implant-Associated Infection Risk. Acta Biomater..

[50] Novikov A.A., Anikushin B.M., Petrova D.A., Konstantinova S.A., Mel’nikov V.B., Vinokurov V.A. (2018). Acid and Oxidative Treatment of Raw Material for the Production of Nanofibrillar Cellulose. Chem. Technol. Fuels Oils.

[51] Cherednichenko K.A., Sayfutdinova A.R., Kraynov A., Anikushin B., Ignatiev V., Rubtsova M.I., Konstantinova S.A., Shchukin D.G., Vinokurov V.A. (2022). A Rapid Synthesis of Nanofibrillar Cellulose/Polystyrene Composite via Ultrasonic Treatment. Ultrason. Sonochem..

[52] Soleymani Eil Bakhtiari S., Bakhsheshi-Rad H.R., Karbasi S., Tavakoli M., Hassanzadeh Tabrizi S.A., Ismail A.F., Seifalian A., RamaKrishna S., Berto F. (2021). Poly(Methyl Methacrylate) Bone Cement, Its Rise, Growth, Downfall and Future. Polym. Int..

[53] Dunne N., Ormsby R.W., Naraghi M. (2011). MWCNT Used in Orthopaedic Bone Cements. Carbon Nanotubes—Growth and Applications.

[54] Ormsby R., McNally T., Mitchell C., Halley P., Martin D., Nicholson T., Dunne N. (2011). Effect of MWCNT Addition on the Thermal and Rheological Properties of Polymethyl Methacrylate Bone Cement. Carbon.

[55] Novikov A.A., Sayfutdinova A.R., Gorbachevskii M.V., Filatova S.V., Filimonova A.V., Rodrigues-Filho U.P., Fu Y., Wang W., Wang H., Vinokurov V.A. (2022). Natural Nanoclay-Based Silver–Phosphomolybdic Acid Composite with a Dual Antimicrobial Effect. ACS Omega.

[56] Meyer J., Piller G., Spiegel C.A., Hetzel S., Squire M. (2011). Vacuum-Mixing Significantly Changes Antibiotic Elution Characteristics of Commercially Available Antibiotic-Impregnated Bone Cements. J. Bone Jt. Surg..

[57] Funk G.A., Burkes J.C., Cole K.A., Rahaman M.N., McIff T.E. (2018). Antibiotic Elution and Mechanical Strength of PMMA Bone Cement Loaded with Borate Bioactive Glass. J. Bone Jt. Infect..

[58] Meeker D.G., Cooper K.B., Renard R.L., Mears S.C., Smeltzer M.S., Barnes C.L. (2019). Comparative Study of Antibiotic Elution Profiles from Alternative Formulations of Polymethylmethacrylate Bone Cement. J. Arthroplast..

[59] Gao S., Lv Y., Yuan L., Ren H., Wu T., Liu B., Zhang Y., Zhou R., Li A., Zhou F. (2019). Improved Bone Ingrowth of Tricalcium Phosphate Filled Poly(Methyl Methacrylate) (PMMA) Bone Cements in Vivo. Polym. Test..

[60] Kim S.B., Kim Y.J., Yoon T.L., Park S.A., Cho I.H., Kim E.J., Kim I.A., Shin J.-W. (2004). The Characteristics of a Hydroxyapatite–Chitosan–PMMA Bone Cement. Biomaterials.

[61] Wekwejt M., Moritz N., Świeczko-Żurek B., Pałubicka A. (2018). Biomechanical Testing of Bioactive Bone Cements—A Comparison of the Impact of Modifiers: Antibiotics and Nanometals. Polym. Test..

[62] Wekwejt M., Chen S., Kaczmarek-Szczepańska B., Nadolska M., Łukowicz K., Pałubicka A., Michno A., Osyczka A.M., Michálek M., Zieliński A. (2021). Nanosilver-Loaded PMMA Bone Cement Doped with Different Bioactive Glasses—Evaluation of Cytocompatibility, Antibacterial Activity, and Mechanical Properties. Biomater. Sci..

[63] Wekwejt M., Etmańska D., Halman A., Pałubicka A., Świeczko-Żurek B., Gajowiec G. (2020). Implant System for Treatment of the Orbital Floor Defects of Blowout Fractures in the Maxillofacial Region Using Polypropylene Yarn and Bioactive Bone Cement. J. Biomed. Mater. Res..

[64] Dallas P., Sharma V.K., Zboril R. (2011). Silver Polymeric Nanocomposites as Advanced Antimicrobial Agents: Classification, Synthetic Paths, Applications, and Perspectives. Adv. Colloid Interface Sci..

[65] Whitehouse M.R., Evans S.L. (2010). Bone Cement: An Overview. IJNBM.

[66] Robu A., Antoniac A., Ciocoiu R., Grosu E., Rau J.V., Fosca M., Krasnyuk I.I., Pircalabioru G.G., Manescu (Paltanea) V., Antoniac I. (2022). Effect of the Antimicrobial Agents Peppermint Essential Oil and Silver Nanoparticles on Bone Cement Properties. Biomimetics.

[67] Tuna E.B., Rohlig B.G., Sancakli E., Evlioglu G., Gencay K. (2013). Influence of Acrylic Resin Polymerization Methods on Residual Monomer Release. J. Contemp. Dent. Pract..

[68] Patel R., Mcconaghie G., Webb J., Laing G., Roach R., Banerjee R. (2023). An Overview of Bone Cement: Perioperative Considerations, Complications, Outcomes and Future Implications. J. Perioper. Pract..

[69] Frew N.M., Cannon T., Nichol T., Smith T.J., Stockley I. (2017). Comparison of the Elution Properties of Commercially Available Gentamicin and Bone Cement Containing Vancomycin with ‘Home-Made’ Preparations. Bone Jt. J..

[70] Funk G.A., Menuey E.M., Ensminger W.P., Kilway K.V., McIff T.E. (2021). Elution of Rifampin and Vancomycin from a Weight-Bearing Silorane-Based Bone Cement. Bone Jt. Res..

[71] Al Thaher Y., Alotaibi H.F., Yang L., Prokopovich P. (2021). PMMA Bone Cement Containing Long Releasing Silica-Based Chlorhexidine Nanocarriers. PLoS ONE.

[72] Ayre W.N., Denyer S.P., Evans S.L. (2014). Ageing and Moisture Uptake in Polymethyl Methacrylate (PMMA) Bone Cements. J. Mech. Behav. Biomed. Mater..

[73] Nelson R.C., Hoffman R.O., Burton T.A. (1978). The Effect of Antibiotic Additions on the Mechanical Properties of Acrylic Cement. J. Biomed. Mater. Res..

[74] Klekamp J., Dawson J.M., Haas D.W., DeBoer D., Christie M. (1999). The Use of Vancomycin and Tobramycin in Acrylic Bone Cement. J. Arthroplast..

[75] Lewis G., Janna S. (2006). Estimation of the Optimum Loading of an Antibiotic Powder in an Acrylic Bone Cement: Gentamicin Sulfate in SmartSet HV. Acta Orthop..

[76] Brock H.S., Moodie P.G., Hendricks K.J., McIff T.E. (2010). Compression Strength and Porosity of Single-Antibiotic Cement Vacuum-Mixed with Vancomycin. J. Arthroplast..

[77] Musib M., Jones J., Chakote K., Hayes W., Saha S. (2012). Microhardness of Bi-Antibiotic-Eluting Bone Cement Scaffolds. Prog. Biomater..

[78] Lilikakis A., Sutcliffe M.P.F. (2009). The Effect of Vancomycin Addition to the Compression Strength of Antibiotic-Loaded Bone Cements. Int. Orthop. (SICOT).

[79] Sanz-Ruiz P., Paz E., Abenojar J., Carlos Del Real J., Vaquero J., Forriol F. (2014). Effects of Vancomycin, Cefazolin and Test Conditions on the Wear Behavior of Bone Cement. J. Arthroplast..

[80] Pithankuakul K., Samranvedhya W., Visutipol B., Rojviroj S. (2015). The Effects of Different Mixing Speeds on the Elution and Strength of High-Dose Antibiotic-Loaded Bone Cement Created with the Hand-Mixed Technique. J. Arthroplast..

[81] Anagnostakos K., Kelm J. (2009). Enhancement of Antibiotic Elution from Acrylic Bone Cement. J. Biomed. Mater. Res..

[82] Shojaeiarani J., Bajwa D.S., Chanda S. (2021). Cellulose Nanocrystal Based Composites: A Review. Compos. Part C Open Access.

[83] Cherednichenko K., Kopitsyn D., Batasheva S., Fakhrullin R. (2021). Probing Antimicrobial Halloysite/Biopolymer Composites with Electron Microscopy: Advantages and Limitations. Polymers.

