# Supplementary material for: Composite Bone Cements with Enhanced Drug Elution
Source: Polymers (Basel). 2023 Sep 14;15(18):3757. doi: 10.3390/polym15183757 (PMC10535863; doi:10.3390/polym15183757)
Supplement: Supplementary file 1 [file polymers-15-03757-s001.zip › polymers-2521476-supplementary.pdf]

polymers

Supplementary Materials
Composite Bone Cements with Enhanced Drug Elution

Kirill Cherednichenko [1], Adeliya Sayfutdinova [1], Denis Rimashevskiy [1,2], Birzhan Malik [3], Andrey Panchenko [1], Maria Kopitsyna [4], Stanislav Ragnaev [5], Vladimir Vinokurov [1], Denis Voronin [1] and Dmitry Kopitsyn [1,*]

[1] Department of Physical and Colloid Chemistry, Faculty of Chemical and Environmental Engineering, National University of Oil and Gas "Gubkin University", Moscow 119991, Russia; cherednichenko.k@gubkin.ru (K.C.)
[2] Department of Traumatology and Orthopedics, Peoples' Friendship University of Russia, Moscow 117198, Russia
[3] Astana Medical University, Beybitshilik Street 49a, Astana 010000, Kazakhstan; bmalik1971@mail.ru
[4] Russian Institute for Scientific and Technical Information "VINITI RAS", Moscow 125190, Russia; mariankuznetsova@gmail.com
[5] Multidisciplinary Hospital Named after Professor Kh.Zh. Makazhanov, Karaganda 100000, Kazakhstan; stas.rangaev@mail.ru
* Correspondence: kopicin.d@inbox.ru

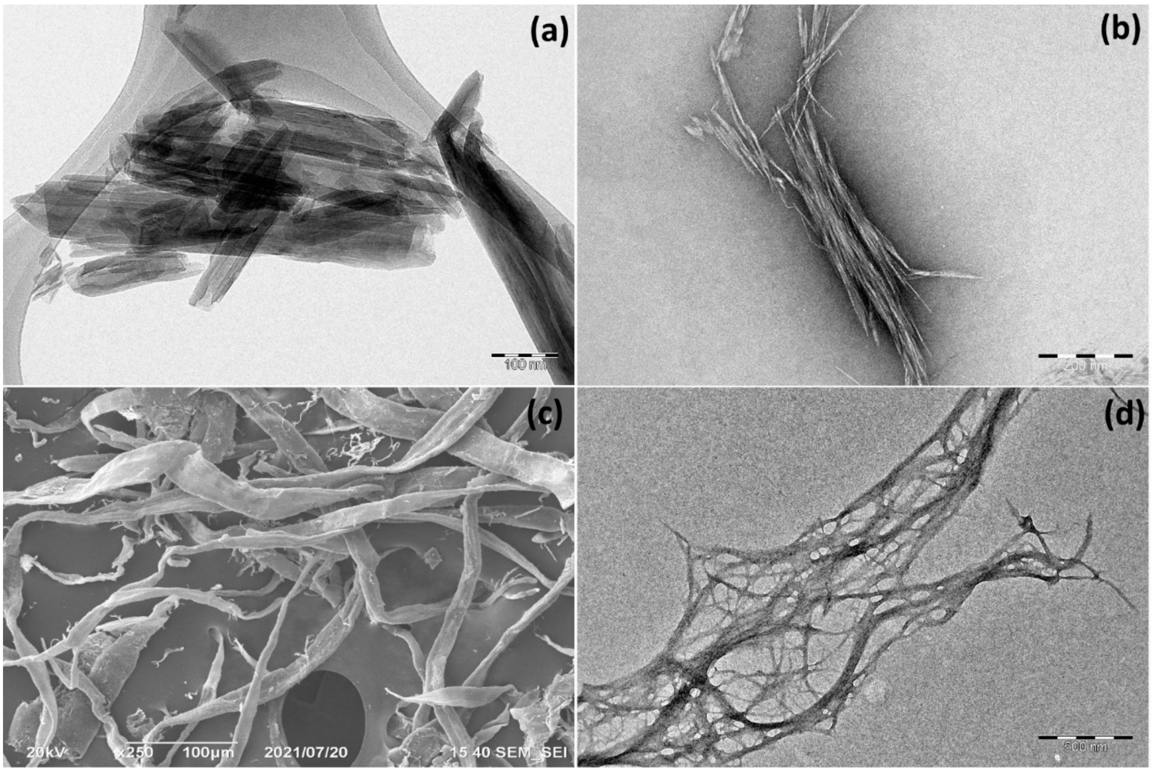

Figure S1. Electron microscopy micrographs of HNT (a), NCC (b), MFC (c), NFC (d).

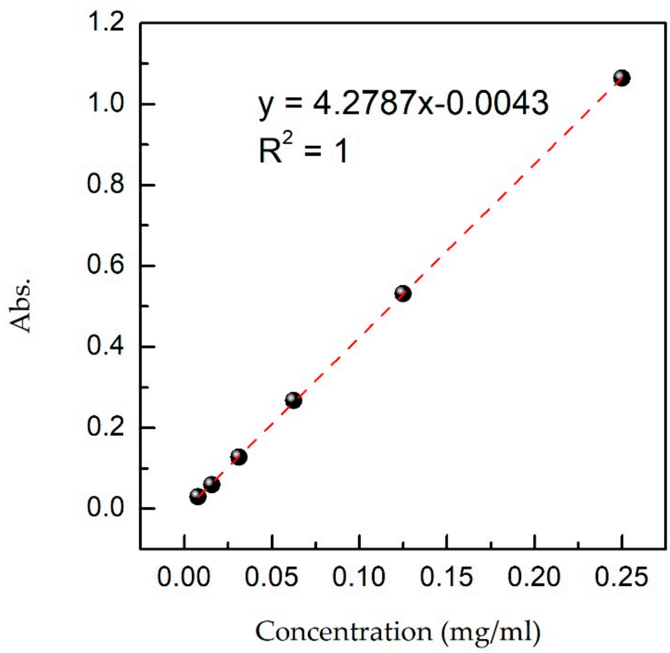

Figure S2. Calibration curve for determination of vancomycin concentration in aqueous solution by its optical density.

Table S1. Composition of surgical Simplex P radiopaque bone cement.

Powder component (40 г)	
MMA and styrene copolymer (1.7 wt.%)	29.49 g
PMMA	6 g
Benzoyl peroxide	0.51 g
$BaSO_4$ (Ph. Eur., USP)	4 g
Liquid component (20 ml, ρ = 0.935 mg/ml)	
MMA	19.5 ml
N,N- dimethylparatoluidine	0.5 ml
Hydroquinone	1.5 mg

Table S2. Composition of surgical Simplex P radiopaque bone cement.

Wood	Spruce
Whiteness, %	88
Ash content, %	0.10
Roughness, μg	107
Fiber length, mm	2.34
pH of the water extraction	5.5 - 7
